# A novel glycogene-related signature for prognostic prediction and immune microenvironment assessment in kidney renal clear cell carcinoma

**DOI:** 10.1080/07853890.2025.2495762

**Published:** 2025-05-07

**Authors:** Xuyan Zhao, Hanxiao Cui, Mingjing Zhou, Xueting Ren, Zihao Li, Peinan Liu, Danni Zhao, Shuai Lin, Huafeng Kang

**Affiliations:** a The Comprehensive Breast Care Center, The Second Affiliated Hospital of Xi’an Jiaotong University, Xi’an, Shaanxi, China; b Department of Neurosurgery, The First Affiliated Hospital of Xi’an Jiaotong University, Xi’an, Shaanxi, China; c Department of Urology, The Second Affiliated Hospital of Xi’an Jiaotong University, Xi’an, Shaanxi, China

**Keywords:** Glycogene, signature, KIRC, immune microenvironment, prognosis

## Abstract

**Background:**

Kidney Renal Clear Cell Carcinoma (KIRC) is a prevalent urinary malignancies worldwide. Glycosylation is a key post-translational modification that is essential in cancer progression. However, its relationship with prognosis, tumour microenvironment (TME), and treatment response in KIRC remains unclear.

**Method:**

Expression profiles and clinical data were retrieved from The Cancer Genome Atlas and Gene Expression Omnibus databases. Consensus clustering, Cox regression, and LASSO regression analyses were conducted to develop an optimal glycogene-related signature. The prognostic relevance of this molecular signature was rigorously analyzed, along with its connections to tumour microenvironment (TME), tumour mutation burden, immune checkpoint activity, cancer-immunity cycle regulation, immunomodulatory gene expression patterns, and therapeutic response profiles. Validation was performed using real-world clinical specimens, quantitative PCR (qPCR), and immunohistochemistry (IHC), supported by cohort analyses from the Human Protein Atlas (HPA) database.

**Results:**

A glycogene-associated prognostic scoring system was established to categorize patients into risk-stratified subgroups. Patients in the high-risk cohort exhibited significantly poorer survival outcomes (*p* < 0.001). By incorporating clinicopathological variables into this framework, we established a predictive nomogram demonstrating strong calibration and a concordance index (C-index) of 0.78. The high-risk subgroup displayed elevated immune infiltration scores (*p* < 0.001), upregulated expression of immune checkpoint-related genes (*p* < 0.05), and an increased frequency of somatic mutations (*p* = 0.043). The risk score positively correlated with cancer-immunity cycle activation and immunotherapy-related signals. The high-risk groups also showed associations with T cell exhaustion, immune-activating genes, chemokines, and receptors. Drug sensitivity analysis revealed that low-risk patients were more sensitive to sorafenib, pazopanib, and erlotinib, whereas high-risk individuals responded better to temsirolimus (*p* < 0.01). qPCR and IHC analyses consistently revealed distinct expression patterns of MX2 and other key genes across the risk groups, further corroborated by the HPA findings.

**Conclusion:**

This glycogene-based signature provides a robust tool for predicting prognosis, TME characteristics, and therapeutic responses in KIRC, offering potential clinical utility in patient management.

## 1. Introduction

Renal cell carcinoma (RCC) is among the top three most prevalent urinary system cancers, making up 2-3% of global cancer cases [1]. According to the most recent report published by the International Agency for Research on Cancer, in 2020, an estimated 400,000 new cases and 180,000 deaths related to RCC were reported [2]. It is estimated that 70% of RCC cases are Clear cell renal cell carcinoma (KIRC), the most frequent histological types [3]. Notably, approximately 30% of RCC patients present with distant metastases at diagnosis [4]. Metastatic KIRC is associated with poor prognosis owing to its inherent resistance to chemotherapy and radiotherapy [5]. For non-metastatic KIRC cases, surgical intervention remains the first-line therapeutic strategy. While recent innovations in pharmacological therapies and operative techniques have markedly enhanced clinical outcomes, a significant number of individuals with localized KIRC ultimately develop metastatic progression or disease recurrence [6]. Consequently, there exists an imperative necessity to explore novel and effective treatment strategies.

Recent therapeutic advancements in metastatic KIRC have been transformative, driven by the integration of immune checkpoint inhibitors (ICIs) and tyrosine kinase inhibitors (TKIs). Notably, regimens combining PD-1 inhibitors (e.g. pembrolizumab) with VEGFR inhibitors (e.g. axitinib) have shown superior survival outcomes compared to traditional treatment protocols [7]. Despite these advancements, ICIs resistance remains a significant clinical challenge. To enhance long-term disease control, adjuvant immunotherapy has been increasingly explored for its potential to reduce postoperative relapse and enhance survival. A meta-analysis involving over 6000 patients, demonstrated that adjuvant PD-1/PD-L1 inhibitors significantly prolonged relapse-free survival (HR 0.72, 95% CI 0.67-0.78), with consistent benefits across age and gender subgroups [7]. Despite these promising findings, their application in KIRC remains underexplored, and predictive biomarkers are urgently needed to guide patient selection and optimise therapeutic strategies [8]. To improve prognosis prediction and therapeutic decision-making for KIRC, a deeper understanding of its molecular mechanisms is crucial.

Glycobiology has gained increasing attention in cancer research due to its pivotal role in tumour progression and potential for tumour therapy [9,10]. Glycosylation is an enzymatic process responsible for the synthesis and attachment of sugar chains [11,12]. Genes encoding glycosyltransferases, glycosidases, and sulfotransferases play crucial roles in regulating glycosylation [13]. Glycosylation is the most significant post-translational modification of proteins in eukaryotic cells, glycosylation participates in the functional formation of proteins [14,15]. Glycosylation genes are essential for modulating immune recognition by influencing antigen presentation, immune cell signalling, and tumour immune evasion. Abnormal glycosylation or glycogene expression is associated with tumour angiogenesis, cell proliferation, apoptosis, and metastasis, indicating that it may be a potential tumour hallmark. For instance, CA19-9 is a sLeA-type glycan epitope used as a standard serum biomarker of digestive tract malignancies [16]. Through ectopic expression and knockout experiments, Wu et al. observed that GALNT10 promoted hepatoma cell proliferation and apoptosis resistance in a glycosyltransferase-dependent manner [17]. Furthermore, Allam et al. found that MGAT3 is critical in regulating the glycosylation of the Notch receptor, which has therapeutic potential to inhibit tumour growth and recurrence in ovarian epithelial cancer [18]. However, the association between the glycogenes and KIRC remains unclear. However, their clinical significance as biomarkers requires further investigation.

Several prognostic models for KIRC have been established based on gene-related biomarkers, including those associated with pyroptosis [19], immune response [20], and angiogenesis [21]. With the ongoing advancements in precision oncology, molecular signatures are increasingly recognized as critical tools for guiding treatment selection [7]. We identified and validated key glycobiology-related genes from The Cancer Genome Atlas (TCGA) and Gene Expression Omnibus (GEO) databases, performed risk stratification, and predicted prognosis. Furthermore, we analysed the correlations between the glycogene-related signature and various factors such as the tumour microenvironment (TME), tumour mutation burden (TMB), immune checkpoint genes, immunoregulatory genes, and drug sensitivity. Our findings clarify the role of glycobiology in KIRC and underscore its potential as a prognostic biomarker and therapeutic target, providing a foundation for precision oncology approaches.

## 2. Methods

### 2.1. Data collection

**Figure 1.**
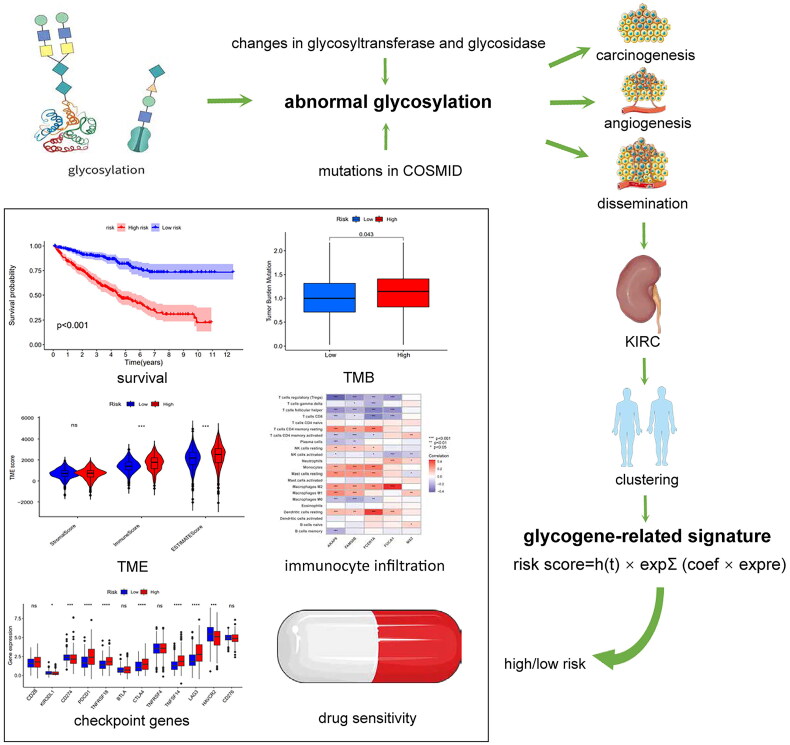
The flow chart of the analysis procedure in this study.

Gene expression and clinical data of 530 patients with KIRC were downloaded from the TCGA database (https://portal.gdc.cancer.gov/). The information on 72 normal cases were also collected. Additional publicly available microarray data and clinical information were obtained from the GEO database (GSE29609, *n* = 39) (https://www.ncbi.nlm.nih.gov/geo). The clinical characteristics extracted from both databases included age, grade, TNM stage, survival time, and disease status (Table 1). Additionally, 185 glycogenes were retrieved from the glycogene database (GGDB) (https://acgg.asia/ggdb2/) and previously published studies [22]. The procedure is illustrated in Figure 1.

**Table 1.** Clinical information of the KIRC cohort in this study.

Variable	Entire cohort (*n* = 569)	Training cohort (*n* = 285)	Testing cohort (*n* = 284)
**Age**			
< =65	370(65.03%)	183(64.21%)	187(65.85%)
>65	199(34.97%)	102(35.79%)	97(34.15%)
**Grade**			
G1-G2	254(44.64%)	122(42.81%)	132(46.48%)
G3-G4	307(53.95%)	159(55.79%)	148(52.11%)
GX	8(1.41%)	4(1.40%)	4(1.41%)
**T**			
T1-T2	356(62.57%)	174(61.05%)	182(64.08%)
T3-T4	213(37.43%)	111(38.95%)	102(35.92%)
**N**			
N0	270(47.45%)	133(46.67%)	137(48.24%)
N1-N2	24(4.22%)	12(4.21%)	12(4.23%)
NX	275(48.33%)	140(49.12%)	135(47.54%)
**M**			
M0	445(78.21%)	220(77.19%)	225(79.23%)
M1	92(16.17%)	49(17.19%)	43(15.14%)
MX	32(5.62)	16(5.61%)	16(5.63%)

### 2.2. Somatic mutation analysis

We gathered 336 KIRC somatic mutations from the TCGA database. Using the “maftools” package, we plotted a waterfall to describe the mutation landscape of glycogenes in patients with KIRC. Moreover, copy number variation (CNV) data were retrieved from the UCSC Xena platform, followed by systematic quantification of CNV prevalence across the glycogene set.

### 2.3. Consensus clustering analysis of glycogenes

To examine the biological properties of glycogenes in patients with KIRC, we used the ‘ConsensusClusterPlus’ package to identify different glycogene-related patterns and classify patients into distinct subtypes according to the expression levels of glycogenes. In addition, principal component analysis (PCA) was conducted to validate the reliability of KIRC stratification.

### 2.4. Identification of immune features in different clusters

Gene set variation analysis (GSVA) was implemented *via* the ‘GSVA’ R package to evaluate pathway-specific variations across distinct molecular clusters. Pathway annotation data (‘c2.cp.kegg.v7.4.’) were acquired from the MSigDB repository. Immune cell infiltration heterogeneity among clusters was investigated by quantifying 23 immune-related markers in each kidney renal clear cell carcinoma (KIRC) sample through single-sample gene set enrichment analysis (ssGSEA). Furthermore, we utilised the ‘limma’ package to identify differentially expressed genes (DEGs) following an adjusted *P*-value < 0.05 and a fold change > 1.5. Gene Ontology and Kyoto Encyclopedia of Genes and Genomes (KEGG) enrichment analyses were conducted on DEGs for functional and pathway annotations. The prognostic significance of each DEG was identified using univariate Cox proportional hazard analysis, and the genes with a *P*-value < 0.05 were designated as prognostic DEGs (PDEGs).

### 2.5. Consensus clustering of prognostic DEGs

Based on the PDEGs, we used the ‘ConsensusClusterPlus’ package to perform a consensus clustering analysis of the genomic subtypes and assign the patients to different clusters. Kaplan-Meier analysis and log-rank tests were conducted to compare differences in overall survival. Additionally, a heatmap integrating clinical information and distinct clusters was displayed.

### 2.6. Construction and assessment of the glycogene-related signature

We divided patients with KIRC into training and validation sets in a 1:1 ratio. To construct a model capable of quantifying all the patients, we performed LASSO regression with a minimum λ and identified five PDEGs as the optimum gene signature in the training set. In the training cohort, a glycogene-associated prognostic scoring system was formulated using LASSO regression coefficients and transcriptomic data. The risk score equation was defined as:

$$\text{Risk score}=h_{0}(t)\cdot \;\text{exp}[\sum_{i=1}^{n}\left(\alpha_{i}\times {\text{gene}}_{i}\text{ expression}\right)]$$

All KIRC patients were assigned risk scores and stratified into low- versus high-risk subgroups based on median score thresholds. Prognostic utility was assessed through survival analysis, receiver operating characteristic (ROC) curves, and risk distribution plots. Additionally, correlations between risk scores and clinicopathological parameters were evaluated. To enhance clinical applicability, a multivariate nomogram integrating this molecular signature with established prognostic indicators (age, tumour grade, T stage, and M stage) was developed for individualized outcome prediction in KIRC.

### 2.7. Exploration of the immune-related characteristics in the TME

Initially, the stromal score, immune score, estimate score, and tumour purity were used to determine the patient’s overall immune status [23]. We used CIBERSORT to visualise the profiles of 22 immunocytes in the KIRC samples [24]. In order to ascertain the function of this risk score in regulating tumour immunity, we analysed the expression levels of 12 checkpoint genes and immunocyte infiltration between the groups. Additionally, the correlation between the risk score and the cancer-immunity cycle (CICs), immunotherapy-associated signals, and TMB was evaluated.

### 2.8. Comparisons of drug sensitivity between two groups

To determine the drug sensitivity in different risk groups, we analysed four common targeted drugs (sorafenib, pazopanib, erlotinib, and temsirolimus) for KIRC using the “pRRophetic” package. This package, developed from cancer cell lines in the Cancer Genome Project, predicts drug sensitivity based on gene expression data [25].

### 2.9. Statistical analysis

The Wilcoxon test was used to compare the differences between the two groups. Kaplan-Meier analysis and log-rank tests were used to explore survival variations between different groups. Univariate and multivariate Cox regression analyses were used utilized to identify prognostic factors. Statistical analysis and plotting in the bioinformatics section were performed using R software (version 4.0.3), with a p-value < 0.05 considered statistically significant. Data analysis for the qPCR section was conducted using SPSS software version 28.0 (SPSS, Chicago, Illinois, USA).

### 2.10. qPCR analysis

Postoperative pathology confirmed 18 cases of KIRC. The patients’ medical histories, preoperative auxiliary examination results, and postoperative pathological findings validated the diagnosis of KIRC. Informed consent forms were signed by the patients and their family members granting permission for participation. Following the risk stratification guidelines defined by the International Metastasis Detection Consortium, the 18 enrolled cases were stratified into intermediate- and low-risk categories, with comprehensive clinical documentation systematically curated. For quantitative PCR (qPCR) validation, total RNA was isolated from KIRC tissue specimens using TRIzol reagent (Takara, China). RNA integrity was verified *via* spectrophotometric analysis (NanoDrop), with absorbance ratios (A260/A280) of 1.8–2.0 considered indicative of high-quality RNA. Complementary DNA (cDNA) synthesis was performed with a commercially available reverse transcription kit (Thermo Fisher Scientific) adhering to standardized protocols. qPCR was conducted using the SYBR Green Master Mix (Diyibio, China) on a qPCR system (Bio-Rad, USA), with each sample run in triplicate to ensure accuracy. The thermal cycling conditions were set as follows: an initial denaturation at 95 °C for 5 min, followed by 40 cycles of 95 °C for 15 s and 60 °C for 30 s. GAPDH was used as the endogenous control for normalisation, and relative gene expression was calculated using the 2^-ΔΔct^ method. The primer sequences were shown as follows:

MX2-F: 5’-CAGAGGCAGCGGAATCGTAA3’
MX2-R: 5’-TGAAGCTCTAGCTCGGTGTTC3’
FAM50B-F: 5’-AAGAGGTTCTCGGCGCATTAC3’
FAM50B-R: 5’-CGGGCCTTCATGTCGTTCA3’
FUCA1-F: 5’-GAAGCCAAGTTCGGGGTGTT3’
FUCA1-R: 5’-GGGTAGTTGTCGCGCATGA3’
AKAP6-F: 5’-AAGAGGCAATGAAGGACATGG3’
AKAP6-R: 5’-TCCTGCTGGACTGAATAGGTTA3’
FCER1A-F: 5’-GTTCTTCGCTCCAGATGGC3’
FCER1A-R: 5’-TTGTGGAACCATTTGGTGGAA3’

### 2.11. Immunohistochemical validation

Paraffin-embedded KIRC tissues from 9 medium-risk and 9 low-risk cases were sectioned into 4-μm slices. The sections were deparaffinization using xylene (5 min × 2) followed by rehydration in graded ethanol (100%, 95%, 85%, 75%) and distilled water. Antigen retrieval was conducted in citrate buffer (**120 °C**, 2 min), and endogenous peroxidase activity was blocked with 3% H**_2_**O**_2_**. Sections were incubated with 5% goat serum to block nonspecific binding, followed by primary antibodies: MX2 (Sanying Biotechnology, China, Cat#13278-1-AP, 1:200 dilution), FAM50B (ABclonal, China, Cat#A20245, 1:150), FUCA1 (ABclonal, China, Cat#A14533, 1:150), AKAP6 (ABclonal, China, Cat#A12798, 1:150), and FCER1A (ABclonal, China, Cat#A1751, 1:150). After PBS washes, HRP-conjugated secondary antibodies (ZSGB-BIO, China) were applied (**37 °C**, 30 min). DAB chromogen (ZSGB-BIO, China) and hematoxylin counterstaining (Solarbio) were performed, with dehydration through graded ethanol and xylene before mounting in neutral resin.

## 3. Results

### 3.1. Genetic variation of glycogenes in KIRC

**Figure 2.**
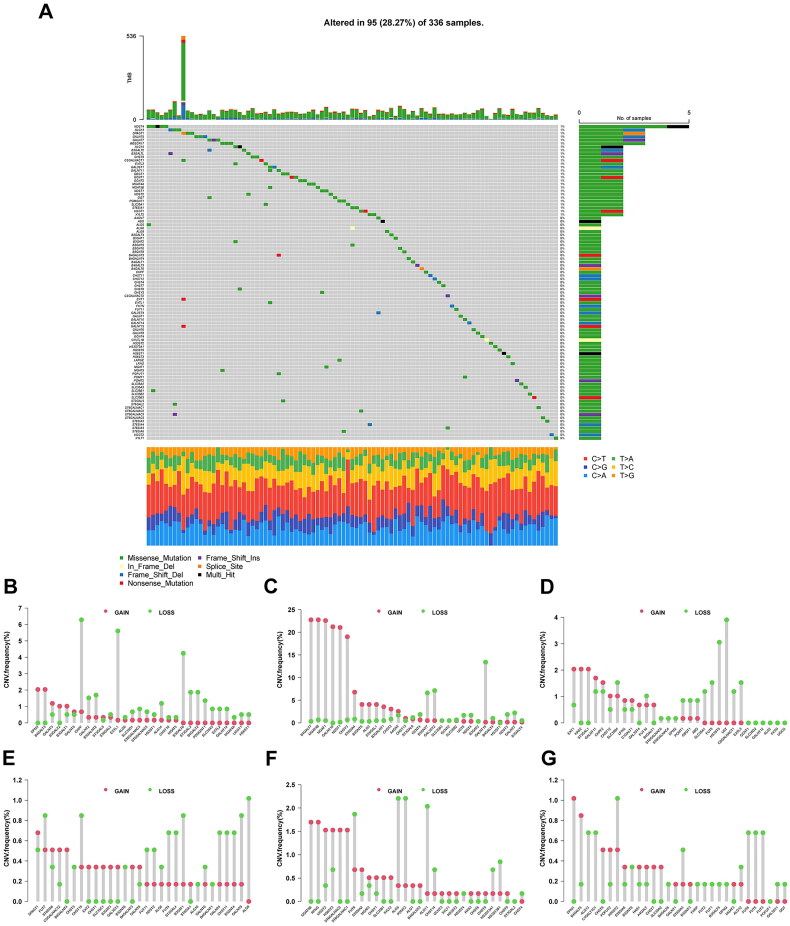
The mutation landscape of glycogenes in KIRC. (A) The mutation landscape of glycogenes in 336 patients with KIRC from the TCGA database. (B-G), the copy number variation frequency of glycogenes. red circle, amplification of the copy number; green circle, loss of the copy number.

Using data obtained from the GGDB Database and previous publications, we identified 185 glycogenes. Genetic variation analysis revealed that 95 (28.27%) of the 336 samples exhibited mutations, with missense mutations being the most frequent. The top five mutated glycogenes were NDST4, ALG13, DPAGT1, GALNT5, and GALNT7 (Figure 2(A)). Copy number variations of the glycogenes on their corresponding chromosomes are shown in Supplementary Figure 1. Additionally, both copy number amplification and loss were common to these glycogenes. Copy number alterations, including amplifications and deletions, were prevalent among these glycogenes. The most frequently amplified loci included B4GALT7, MGAT4B, and MGAT1, while recurrent deletions predominantly affected GALNT15, GAL3ST2, and CHPF (as illustrated in Figure 2(B–G)).

### 3.2. Comparison of glycogene expression between normal and KIRC tissues

**Figure 3.**
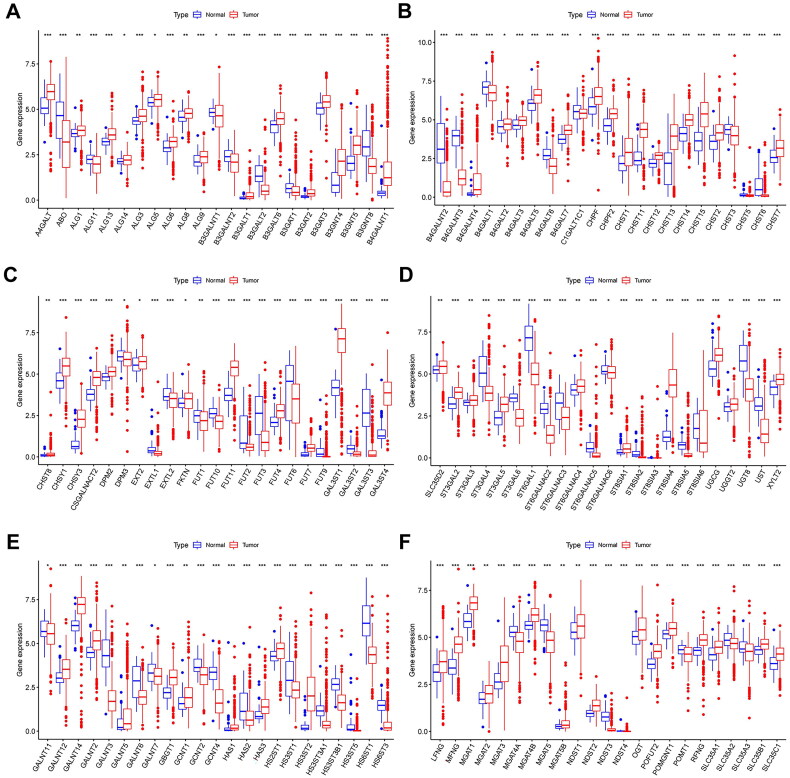
The differential expression of glycogenes between KIRC and adjacent normal tissues (*p < 0.05, **p < 0.01, ***p < 0.001).

To investigate the biological significance of the 185 identified glycogenes in KIRC, we first compared their expression levels in 530 KIRC and 72 adjacent normal samples from the TCGA database. The differential analysis results indicated that the expression of 138 glycogenes in KIRC was markedly different from that in normal tissues, comprising 58 downregulated and 80 upregulated genes (Figure 3(A–F), Supplementary Table 1) (*p* < 0.05).

### 3.3. Subtype identification through consensus clustering of glycogenes in KIRC

**Figure 4.**
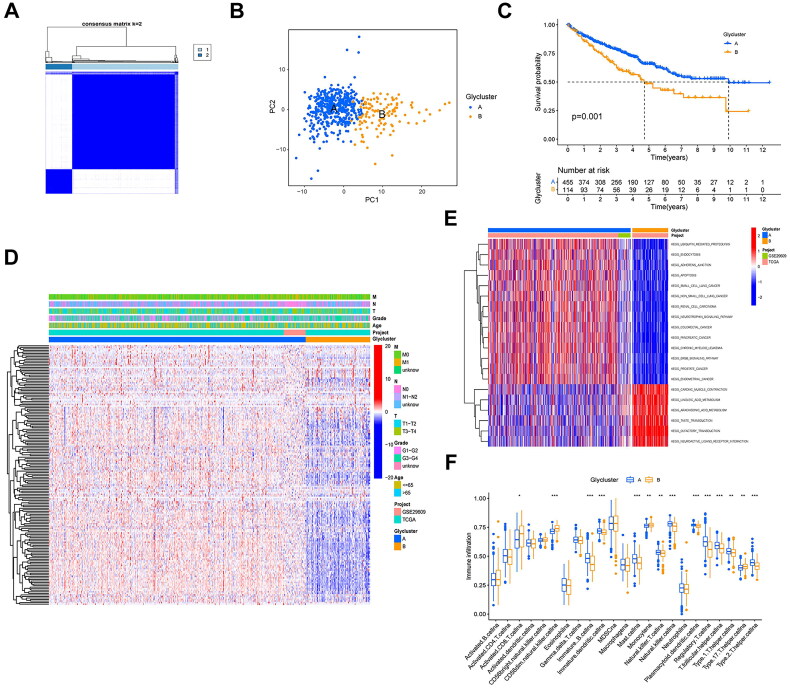
Identification of glycogene-related clusters in patients with KIRC. (A), the consensus clustering analysis based on the expression levels of glycogenes in patients with KIRC (TCGA-KIRC and GSE29609). (B), the distribution of glycogene expression in distinct clusters. (C), the Kaplan-Meier curve of overall survival in different clusters. (D), the heatmap integrating the glycogenes and clinical characteristics in different clusters. (E), GSVA enrichment analysis in distinct clusters. (F), the differential expression of the immunocytes in distinct clusters.

Following the integration of TCGA and GEO datasets, a consensus clustering methodology was applied to stratify KIRC patients based on glycogene expression profiles. This analysis delineated two molecular subgroups: Cluster A (*n* = 455) and Cluster B (*n* = 114), as visualized in Figure 4(A). Principal component analysis (PCA) revealed marked divergence in transcriptional landscapes between the subgroups (Figure 4(B)). Subsequent investigations identified significant associations between cluster membership and clinicopathological parameters (Figures 4(C,D)), with Cluster A demonstrating superior overall survival outcomes (*p* = 0.001). To elucidate biological distinctions between clusters, transcriptomic heterogeneity was explored *via* Gene Set Variation Analysis (GSVA). Cluster A exhibited pronounced enrichment in pathways related to ubiquitin-dependent proteolysis, adherens junction regulation, apoptosis, renal carcinogenesis, and ERBB signalling. Conversely, Cluster B displayed preferential activation of metabolic pathways, including linoleic acid and arachidonic acid metabolism, alongside neuroactive ligand-receptor interactions (Figure 4(E)). Immune profiling further differentiated the subgroups, as shown in Figure 4(F). Cluster A exhibited elevated infiltration of immunologically active populations, such as immature B cells, dendritic cell precursors, mast cells, and natural killer (NK) cells (*p* < 0.05). In cluster B, a higher infiltration rate was observed in activated CD8 + T cells, CD56dim NK cells, monocytes, and type 17 helper T cells (*p* < 0.05). According to the above analyses, a marked differentiation was observed between the two clusters, indicating the presence of DEGs. We then identified 6122 DEGs between the two clusters and subsequently performed Gene Ontology and KEGG enrichment analyses. The enriched biological processes included ameboid-type cell migration, positive regulation of cell adhesion, and cell-substrate adhesion. The enriched cellular components included focal adhesions, cell-substrate junctions, and cell-cell junctions. The significantly enriched molecular functions included actin binding, cadherin binding, and protein serine/threonine kinase activity. KEGG analysis revealed that the DEGs were mainly associated with the PI3K-Akt pathway, MAPK pathway, and Salmonella infection (Supplementary Figure 2).

### 3.4. Identification of two KIRC clusters via consensus clustering analysis of the prognostic DEGs

**Figure 5.**
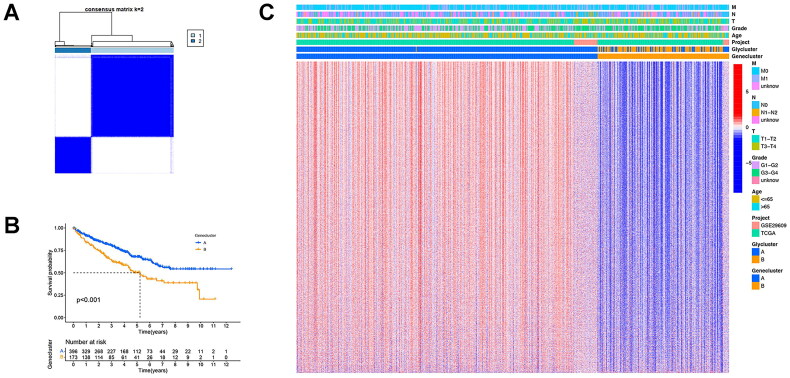
Identification of genomic clusters in patients with KIRC. (A), the consensus clustering analysis of the prognostic DEGs in patients with KIRC (TCGA-KIRC and GSE29609). (B), the Kaplan-Meier curve of overall survival in distinct genomic clusters. (C), the heatmap integrating prognostic DEGs and clinical characteristics in different clusters.

To discern prognosis-associated differentially expressed glycogenes (PDEGs), a univariate Cox proportional hazards model was implemented. Molecular subgroups were subsequently delineated through consensus clustering based on PDEG expression patterns, stratifying KIRC patients into two distinct cohorts: Cluster A (*n* = 396) and Cluster B (*n* = 173), as illustrated in Figure 5(A). Survival analysis *via* Kaplan-Meier curves demonstrated significantly prolonged survival in Cluster A compared to Cluster B (*p* < 0.001; Figure 5(B)). Integrative heatmap visualization of clinical metadata and molecular clusters further revealed pronounced PDEG upregulation in Cluster A (Figure 5(C)).

### 3.5. Construction and validation of the glycogene-related signature in prognosis prediction

**Figure 6.**
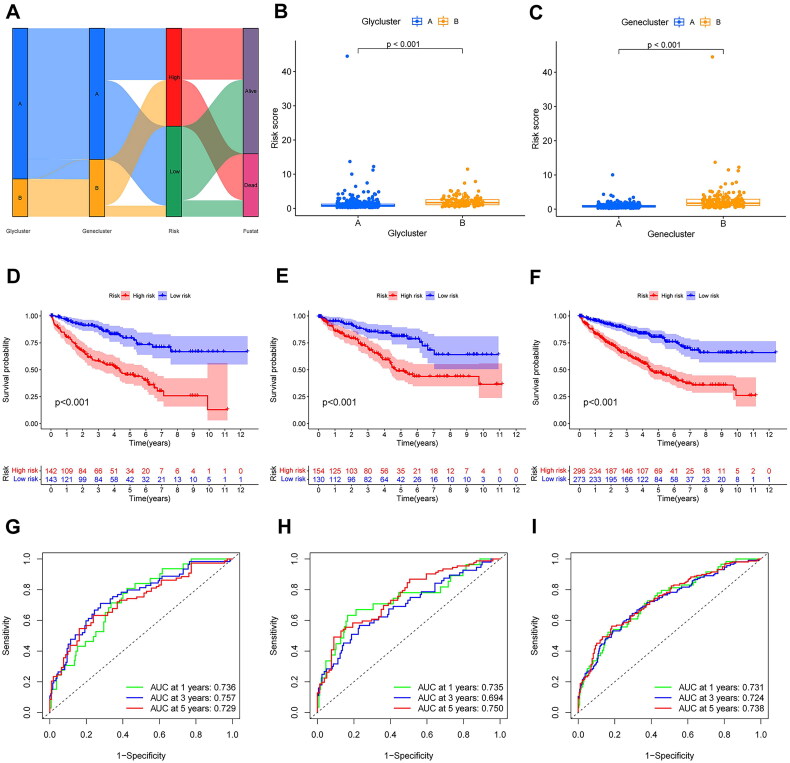
Construction and evaluation of the risk score model. (A), the alluvial diagram showing the procedure of our analysis. (B), the comparison of risk score in different glycogene-related clusters. (C), the comparison of risk score in distinct genomic clusters. The Kaplan-Meier curve of overall survival between high- and low- risk patients (D) in the KIRC training cohort, (E) in the KIRC validation cohort, (F) in the whole KIRC cohort. The time-dependent ROC curves of the risk score model (G), in the KIRC training cohort, (H) in the KIRC validation cohort, (I) in the whole KIRC cohort.

**Figure 7.**
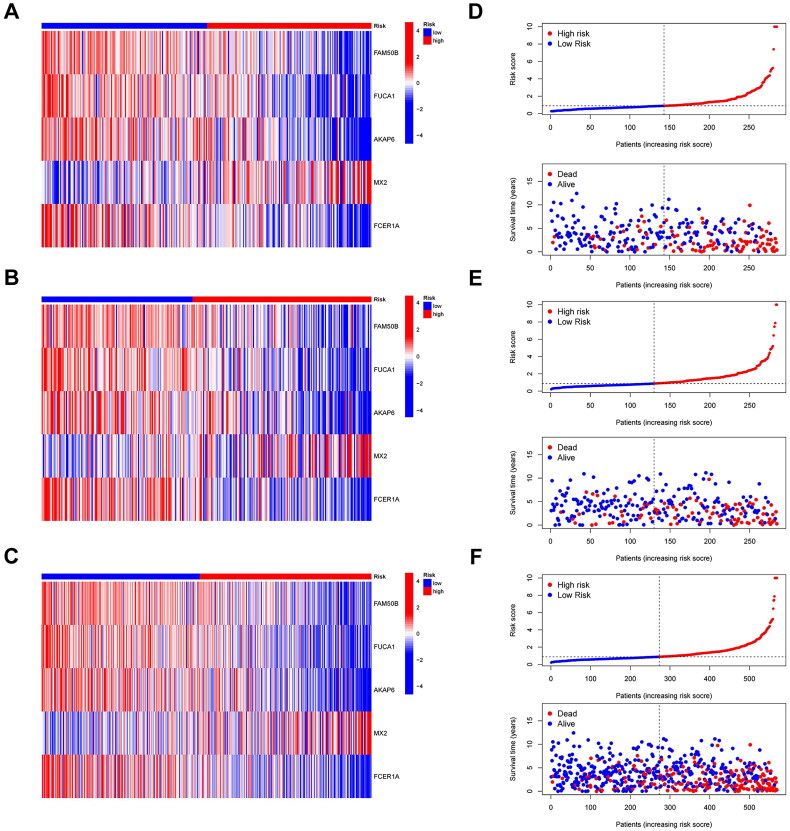
Exploration of the signature. The heatmap demonstrating the expression levels of the glycogene-related signature in different risk groups. (A), in the KIRC training cohort, (B), in the KIRC validation cohort, (C), in the whole KIRC cohort. Survival status and the distribution of the risk score (D), in the KIRC training cohort, (E), in the KIRC validation cohort, (F), in the whole KIRC cohort.

**Figure 8.**
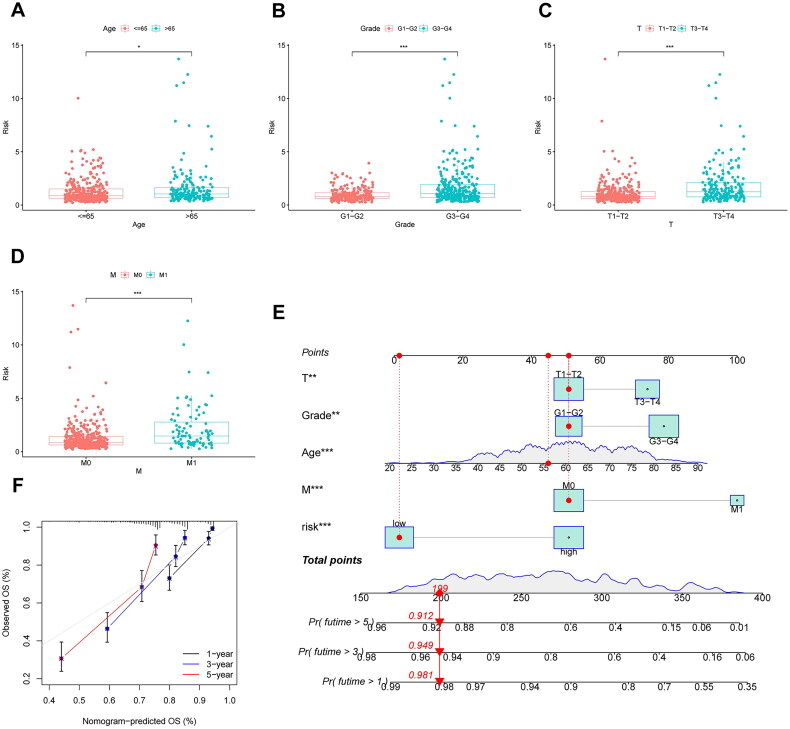
Construction of a nomogram to predict the prognosis of KIRC patients. (A-D), the correlation between risk score and age, grade, T and M stages, respectively. (E) a comprehensive nomogram integrating the risk score model and clinicopathological characteristics. (F), calibration curves of the nomogram to predict survival at 1-, 3- and 5-year intervals.

All subjects with KIRC were randomly separated into training and testing groups in a 1:1 ratio for the further establishment and validation of the signature. Subsequently, we performed LASSO regression and identified five PDEGs to construct a prediction model. The procedure for our analysis is shown in the alluvial diagram (Figure 6(A)). A prognostic risk scoring model was derived from gene expression profiles and regression coefficients, formulated as follows: risk score = h_0_exp [(0.547 × expression of MX2) – (0.215 × expression of FAM50B) – (0.378 × expression of FUCA1) – (0.337 × expression of AKAP6) – (0.178 × expression of FCER1A)]. Patients in glycluster B and gene cluster B had higher risk scores than those in cluster A (*p* < 0.001) (Figure 6(B,C)). We calculated the risk scores of patients in the training group and stratified them into high- and low-risk groups according to the median value. Patients stratified into the high-risk subgroup exhibited significantly inferior survival outcomes compared to their low-risk counterparts (*p* < 0.001; Figure 6(D)). These prognostic disparities remained consistent in both the internal validation cohort and the combined cohort (*p* < 0.001; Figures 6(E,F)). Furthermore, the molecular signature demonstrated limited predictive capacity for survival probabilities at 1, 3, and 5 years, as evidenced by area under the curve (AUC) values (see in Figures 6(G–I)): training cohort: 0.74 (1-year), 0.76 (3-year), 0.73 (5-year); testing cohort: 0.74 (1-year), 0.69 (3-year), 0.75 (5-year); and entire cohort: 0.73 (1-year), 0.72 (3-year), 0.74 (5-year). As shown in the heatmap, MX2 was highly expressed in the high-risk group, whereas the other four genes (FAM50B, FUCA1, AKAP6, and FCER1A) showed the opposite results (Figure 7(A–C)). The survival status and distribution of risk scores are shown in Figure 7(D–F). These analyses identified age, tumour grade, T stage, M stage, and the risk score as independent predictors of clinical outcomes in KIRC. Subsequent evaluation of the interplay between risk scores and these prognostic factors revealed significantly elevated risk scores among older individuals, those with poorly differentiated tumours, advanced local tumour invasion (T stage), and metastatic progression (M stage) (*p* < 0.05; Figures 8(A–D)). Furthermore, we constructed a comprehensive nomogram integrating the risk score and clinicopathological features to predict the survival probability at 1-, 3-, and 5-year intervals (Figure 8(E)). The calibration curves and C-index (0.78) revealed high coherence and accuracy between the predicted and observed overall survival after 1, 3, and 5 years (Figure 8(F)).

### 3.6. Immune signature profiling across risk-stratified cohorts

**Figure 9.**
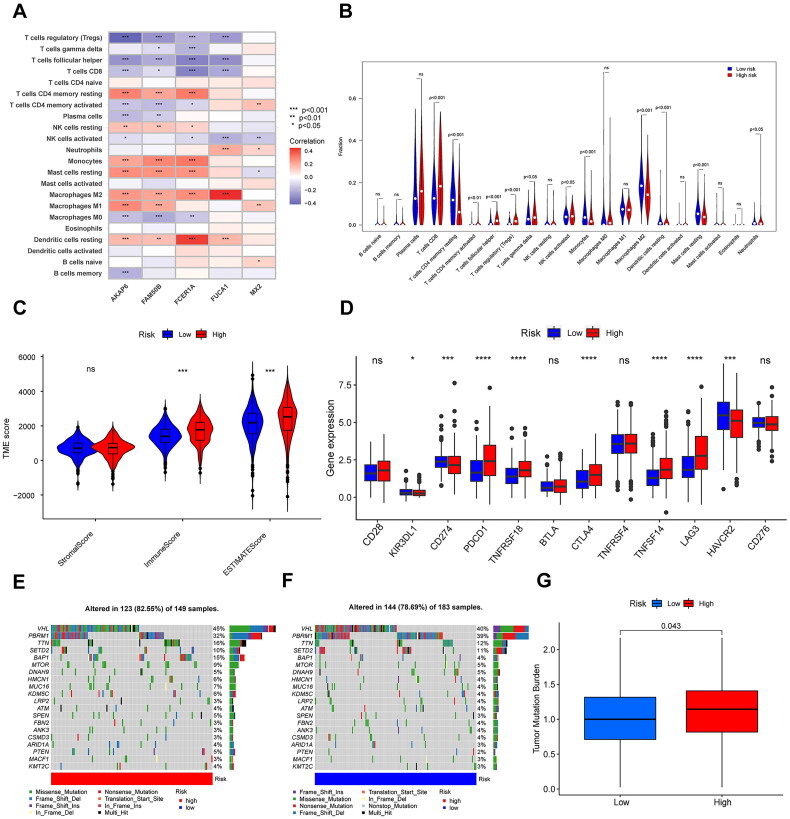
Identification of the immune-related characteristics of the signature. (A), the correlation analysis between the glycogene-related signature and immunocyte infiltration. (B), the differential expression of immune cells in different risk groups. (C), the comparison of TME score between the high- and low-risk groups. (D), the differential expression of checkpoint genes in different risk groups. (E), distribution of the top 20 most frequently mutated genes in patients of the high-risk group. (F), distribution of the top 20 most frequently mutated genes in patients of the low-risk group. (G), the comparison of TMB in different risk groups.

**Figure 10.**
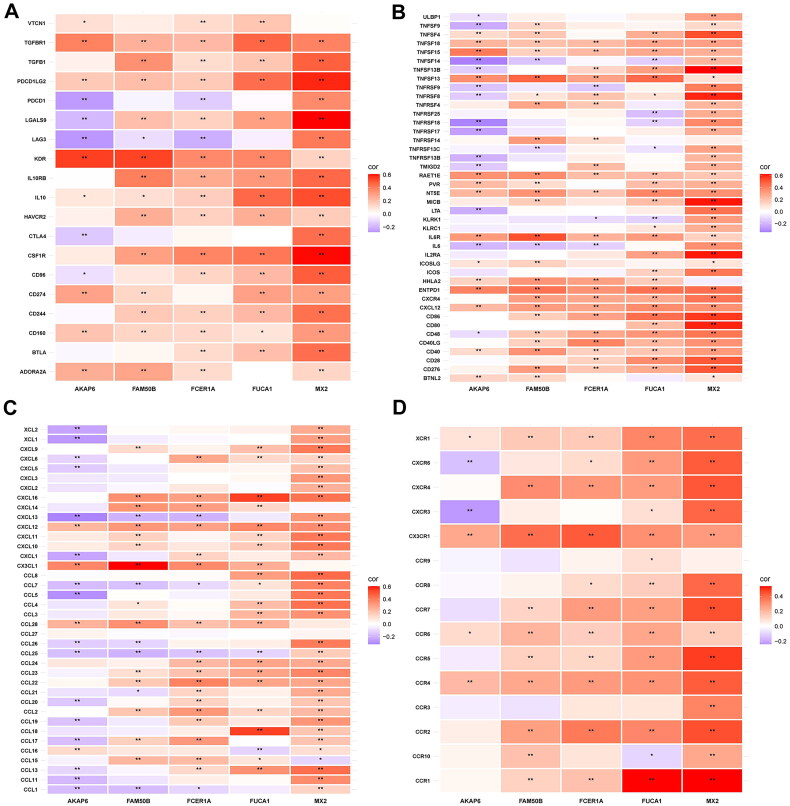
The correlation between the glycogene-related signature and immunoregulation-associated genes. (A), the heatmap demonstrating the correlation between glycogene-associated signature and T cell exhaustion-related genes. (B), the heatmap demonstrating the correlation between the glycogene-related signature and immune activating genes. (C), the heatmap demonstrating the correlation between glycogene-related signature and the chemokines. (D), the heatmap demonstrating the correlation between glycogene-related and the chemokine receptors.

Correlation analyses of the glycogene-related signatures with immunocyte infiltration were conducted to explore the impact of our model on the tumour microenvironment of patients with KIRC (Figure 9(A)), indicating that this signature positively correlated with the infiltration levels of resting dendritic cells, macrophages M1, macrophages M2, monocytes, resting NK cells, and resting CD4 memory T cells. In contrast, the signature was negatively correlated with regulatory T cells, follicular helper T cells, CD8 + T cells, activated NK cells, and macrophages M0 (*p* < 0.05). Distinct immune cell composition was evident between risk-stratified cohorts, with significant disparities observed across 12 immune cell subsets (*p* < 0.05; Figure 9(B)). Using the ESTIMATE algorithm, immune microenvironment scores (including immune and stromal components) were computed, revealing markedly elevated scores in the high-risk cohort compared to the low-risk subgroup (*p* < 0.001; Figure 9(C)). Consistent with prior clinical investigations into immune checkpoint dynamics in KIRC, our analysis demonstrated predominant upregulation of checkpoint-related genes in high-risk individuals (*p* < 0.05; Figure 9(D)). The glycogene-derived risk score exhibited strong positive correlations with key phases of the cancer-immunity cycle (CICs), notably T-cell priming/activation, immunocyte recruitment, and tumour cell recognition (Figure 9(E)). Furthermore, the risk score showed significant associations with pro-immunotherapeutic molecular signatures (Figure 9(F)). To assess genomic instability, somatic mutation landscapes were analyzed across risk cohorts (Figure 9(G)). Among the top 20 mutated genes, 82.55% (123/149) of high-risk samples harboured somatic variants, compared to 78.69% (144/183) in the low-risk group. Tumour mutational burden (TMB) was significantly elevated in high-risk patients (*p* = 0.043). T cell depletion refers to the loss of T cell function in patients with tumours and chronic infections. Notably, significant correlations were observed between the signature and canonical T-cell exhaustion markers PDCD1, LAG3 and CD274 (Figure 10(A)). Similarly, strong linkages emerged with immune-activating factors such as CD276, CD40, and TNFSF18 (Figure 10(B)). Chemotactic signalling pathways, encompassing chemokines and their receptors, also exhibited pronounced associations with the signature (Figures 10(C,D)).

### 3.7. Identification of drug sensitivity among different risk groups

**Figure 11.**
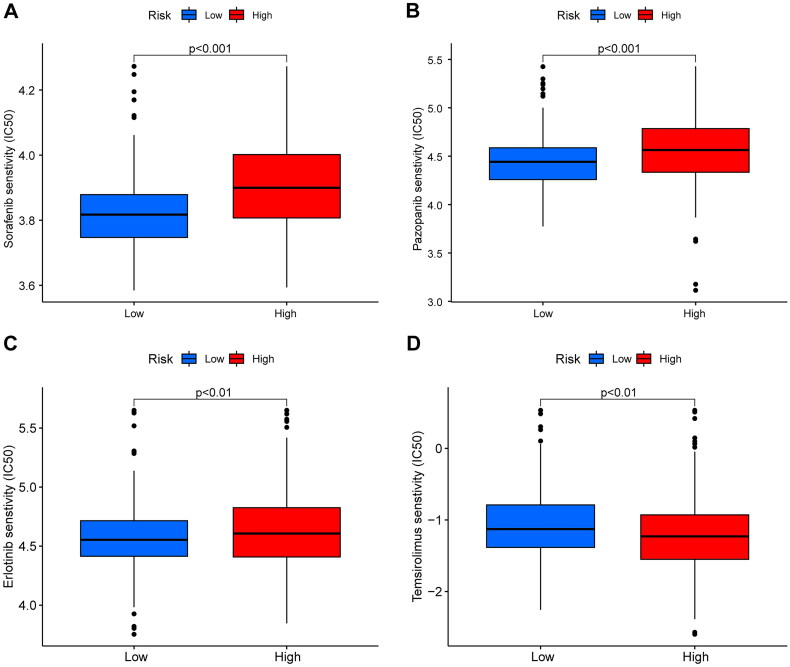
Drug sensitivity analysis between high- and low-risk patients. (A), sorefenib. (B), pazopanib. (C), erlotinib. (D), temsirolimus.

Using the ‘pRRophetic’ package, we explored the drug sensitivity of four common targeted drugs in distinct groups. The results demonstrated that low-risk patients were more sensitive to sorafenib, pazopanib, and erlotinib and less sensitive to temsirolimus (Figure 11(A–D)).

### 3.8. qPCR validation

**Figure 12.**
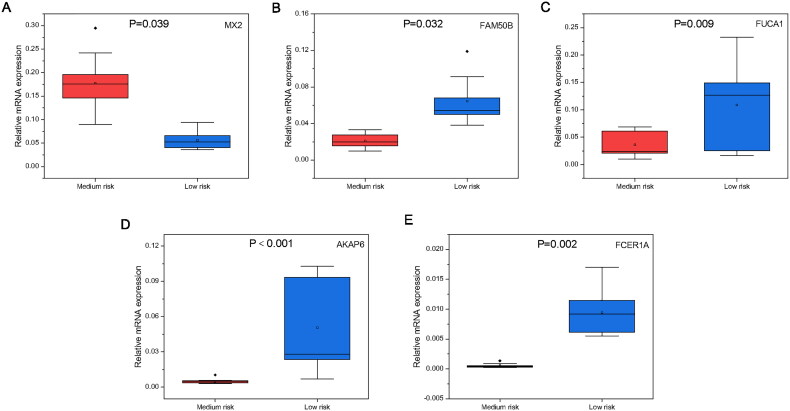
Gene expression levels in the medium-risk and low-risk groups. (A), MX2. (B), FAM50B. (C), FUCA1. (D), AKAP6. (E), FCER1A.

Subsequently, the expression of relevant genes was validated. The expression levels of the five prognostic differential genes identified through risk screening differed significantly between the intermediate-risk group and low-risk group (*p* < 0.05). Notably, MX2 exhibited a significantly higher expression in the medium-risk group than the low-risk group (Figure 12(A)), whereas the other four genes displayed an inverse pattern (Figure 12(B–E)).

### 3.9. Immunohistochemical validation

**Figure 13.**
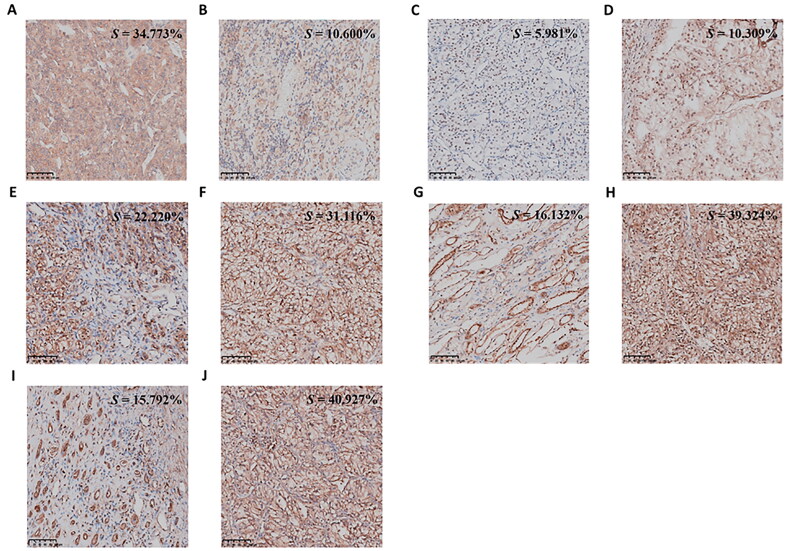
IHC Results and staining intensity. Representative IHC staining of MX2, FAM50B, FUCA1, AKAP6, and FCER1A in medium-risk and low-risk KIRC tissues. (A, B) MX2; (C, D) FAM50B; (E, F) FUCA1; (G, H) AKAP6; (I, J) FCER1A. Panels on the left (A, C, E, G, I) represent medium-risk cases, while panels on the right (B, D, F, H, J) represent low-risk cases. Scale bar = 100 μm.

Protein-level validation corroborated qPCR results, demonstrating marked disparities in biomarker expression between risk-stratified cohorts. Immunohistochemical (IHC) profiling identified cytoplasmic MX2 overexpression in medium-risk specimens (*p* ≤ 0.05; Figure 13(A)), whereas FAM50B, FUCA1, AKAP6, and FCER1A exhibited ­suppressed expression. Conversely, in high-risk patients, FAM50B, FUCA1, AKAP6, and FCER1A displayed ­significantly ­attenuated expression levels relative to the low-risk group (*p* ≤ 0.05; Figures 13(B–E)), with distinct subcellular compartmentalization (cytoplasmic/nuclear). Staining intensity quantification *via* ImageJ software (NIH, USA), calculated as integrated optical density normalized to positively stained regions, validated these expression patterns (all *p* ≤ 0.01), confirming transcriptional-translational concordance of these prognostic biomarkers in KIRC.

## 4. Discussion

Glycosylation, an enzymatic process that catalyses sugar chain synthesis and connection, is crucial in carcinogenesis and tumour development. Recent studies have highlighted the significant effect of glycosylation on cytodifferentiation and immune regulation in various malignancies [26]. Moreover, accumulating evidence indicates that glycosylation disorders may represent potential hallmarks of immunomodulation triggered by tumour progression and metastasis, thereby providing new insights into cancer diagnosis, prognosis, and targeted therapies [27].

KIRC has emerged as an aggressive malignancy of the urinary system and is characterised by increasing incidence and mortality rates. Traditionally, clinicopathological features have been pivotal for predicting the prognosis of KIRC. However, as the molecular mechanisms of KIRC become better understood, molecular risk models have demonstrated significant potential for providing more precise predictions and guiding personalised treatment strategies. Previous models, including those related to pyroptosis and immune mechanisms, have shown reliable prognostic value in patients [19; 20].

We established a glycogene-derived signature for risk stratification, prognosis prediction, and immune characterisation of patients with KIRC. This model effectively differentiated high- and low-risk patients, with higher risk scores associated with more aggressive tumour features and poorer overall survival outcomes. Notably, this signature exhibited significant associations with T-cell exhaustion-related genes, immune-activating genes, chemokines, and chemokine receptors, underscoring its potential for predicting responses to immunotherapy. Moreover, as an independent prognostic factor, the risk score demonstrated excellent sensitivity and specificity. It significantly contributed to a comprehensive nomogram that accurately predicted patient prognosis when integrated with the four clinical variables. Similar to the metastatic status, the risk score is crucial in the overall prognostic assessment, emphasising its clinical relevance in prognosis prediction and immunotherapy guidance for patients with KIRC.

(TME) serves as a pivotal modulator of neoplastic initiation and malignant progression. [28,29]. We observed significant between-group differences in TME. High-risk patients exhibited higher immune scores and poorer prognoses, aligning with findings from previous studies [20]. Furthermore, high-risk patients have increased proportions of CD8+ T cells, activated NK cells, and regulatory T cells, all of which suppress antitumour immunity and contribute to immune dysfunction and tumour progression [30]. CD8+ T and NK cells are pivotal immunocytes that recognize and eradicate cancerous cells [31,32]. However, prolonged exposure to antigens or tumours can lead to the exhaustion of these effector lymphocytes. A previous study confirmed that T-cell exhaustion is the core factor leading to the immunosuppressive characteristics of KIRC [33]. Additionally, tumours may develop more effective immune escape strategies to disrupt their antitumour effects, resulting in poorer survival outcomes. In recent years, some cancers have been observed to respond well to ICB therapy. Transcriptional profiles of therapeutically relevant immune checkpoint genes in solid tumours were comparatively analysed across risk-stratified cohorts [34]. Analytical outcomes revealed significant upregulation of key checkpoint regulators, including PD1, CTLA4, LAG3, TNFSRF14, and TNFRSF18 in high-risk individuals. Several phase III clinical trials have recently demonstrated that ICB-based regimens significantly improve prognosis compared with traditional TKIs, especially in advanced KIRC. The CheckMate 214 trial revealed that integrating the PD-1 inhibitor nivolumab with the CTLA4 inhibitor ipilimumab significantly enhanced overall survival and progression-free survival while causing fewer side effects than first-line sunitinib therapy. This finding offers renewed hope for patients with advanced RCC [35,36]. Furthermore, the CheckMate 025 clinical trial established that nivolumab exhibits superior overall survival outcomes, enhanced safety profiles, and improved tolerability compared to everolimus in individuals diagnosed with KIRC [37]. As a T-cell exhaustion-promoting gene, LAG3 is a potential therapeutic target. The RELATIVITY-047 trial showed that the combined use of a LAG3 inhibitor and nivolumab had remarkable efficacy in untreated advanced melanomas [38]. Therefore, we speculated that LAG3 may be a novel target for treating high-risk patients with KIRC. Additionally, TNFRSF14, a co-inhibitory molecule that inhibits cytokine production and T-cell proliferation, is a promising marker for prolonging survival in patients with malignancies [39]. Further examinations need to confirm the functions of these checkpoint genes in patients with KIRC.

Somatic mutations play crucial roles in carcinogenesis and tumour progression. Notably, a high TMB is associated with increased immunogenic neoantigen formation, which can enhance immune recognition and predict responses to ICB therapy [40,41]. Consequently, high-risk patients may derive greater benefits from ICB treatment than low-risk patients. Furthermore, T cell exhaustion, a hallmark of immune dysfunction in malignancies and chronic infections, is closely linked to the efficacy of immunotherapy. The glycogene-based signature exhibited robust associations with T-cell exhaustion markers, immunostimulatory mediators, and chemotactic signalling components (chemokines or receptors), underscoring its utility in forecasting immunotherapy efficacy. Following systematic validation of this prognostic model, we posit its clinical applicability as a predictive indicator for both survival outcomes and therapeutic responses to immunotherapy in KIRC patients.

This study’s primary strength was the integration of real-world clinical data, which enhanced the validity and translational impact of our findings. Unlike most previous studies that relied primarily on cell line models or animal experiments, we used pathological specimens from patients with varying prognoses to perform qPCR and IHC validation, ensuring a more clinically relevant assessment. Gene expression analysis of KIRC tissue samples confirmed that the glycogene-derived signature accurately predicted survival outcomes. Notably, IHC validation revealed expression patterns consistent with qPCR results, reinforcing the reliability of our findings at the transcriptional and protein levels. The significant upregulation of MX2 in high-risk patients, along with the reduced expression of FAM50B, FUCA1, AKAP6, and FCER1A in this group, further supports their potential roles in tumour progression and immune regulation. Given the complexity of post-transcriptional modifications, protein-level validation is essential for confirming gene expression trends and assessing their biological significance. The consistency between immunohistochemical (IHC) and quantitative PCR (qPCR) data validates the reliability of the candidate biomarkers and the prognostic utility of the glycogene-driven risk stratification framework for enhancing clinical care strategies in KIRC.

Analysis of the Human Protein Atlas (HPA), a publicly available repository documenting tissue- and cellular-level distribution profiles of approximately 24,000 human proteins, further validated our findings. Patients exhibiting elevated MX2 expression levels showed a marked reduction in overall survival, whereas diminished expression of the remaining four genes correlated with prolonged survival outcomes. As depicted in Supplementary Figure s 3 A–3E, cohort stratification into high- and low-expression subgroups (using median expression values as thresholds) revealed statistically significant survival disparities during longitudinal follow-up. While methodological distinctions exist between this external validation approach and our experimental framework, the concordant outcomes substantiate the robustness of our conclusions across independent platforms.

Although the model developed in this study has undergone rigorous cross-validation and experimental verification, several limitations warrant consideration. First, inherent biases in public databases may influence the research findings. Therefore, incorporating additional real-world data could improve the model’s robustness and generalizability. Second, the functional mechanisms of glycosylation-related genes in KIRC remain poorly understood. Future investigations should include functional assays, such as gene knockout and overexpression experiments, to clarify the roles of key glycosylation-related genes in KIRC pathogenesis. Experimental investigations employing murine models may yield critical insights into how genetic modifications influence neoplastic progression and metastatic dissemination, thereby clarifying fundamental molecular pathways.

The glycosylation-related gene-based prognostic model constructed in this study provides a novel biomarker combination strategy for early malignancy screening and prognostic risk assessment in clinical practice. While significant progress has been made in deciphering the molecular mechanisms of glycosylation dysregulation in oncogenesis and metastatic progression [42], translating these insights into clinically actionable risk stratification tools remains constrained by limitations in methodology standardization and validation frameworks. Building on the findings of this study, the next five years are expected to see advancements in the clinical adaptation of glycosylation-targeted therapies, microenvironment heterogeneity analysis guided by spatial transcriptomics, and precision therapy development driven by single-cell proteomics [43]. Integrating these approaches will facilitate a transition from static characterization to dynamic, multidimensional analysis of the tumour microenvironment, driving systematic advancement in precision oncology research.

## 5. Conclusion

The glycogene-related prognostic model established and rigorously validated in this research offers a novel framework for stratifying survival outcomes and characterizing immune microenvironment dynamics in KIRC, potentially advancing personalized therapeutic strategies for this patient population.

## Supplementary Material

Supplementary Material.pdf

## Data Availability

The data that support the findings of this study are available from the corresponding author upon reasonable request.

## Study period

The study period was from September 18th, 2023 to October 12th, 2024.
