# Supplementary material for: A novel glycogene-related signature for prognostic prediction and immune microenvironment assessment in kidney renal clear cell carcinoma
Source: Ann Med. 2025 May 7;57(1):2495762. doi: 10.1080/07853890.2025.2495762 (PMC12064129; doi:10.1080/07853890.2025.2495762)
Supplement: Supplementary Material.pdf [file IANN_A_2495762_SM2374.pdf]

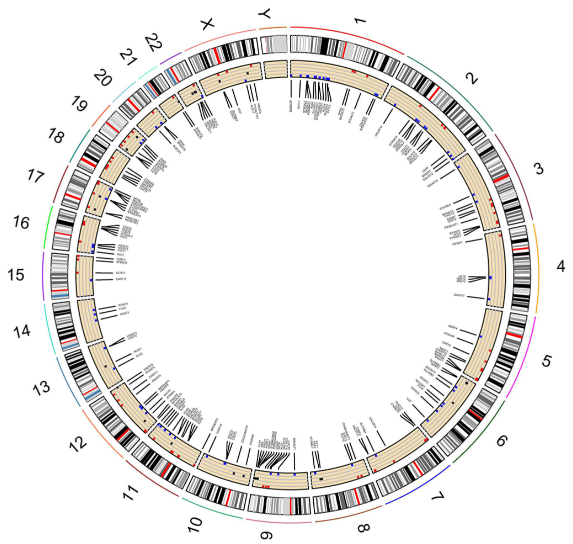

Supplement Figure 1 The copy number variations of glycogenes on the chromosome

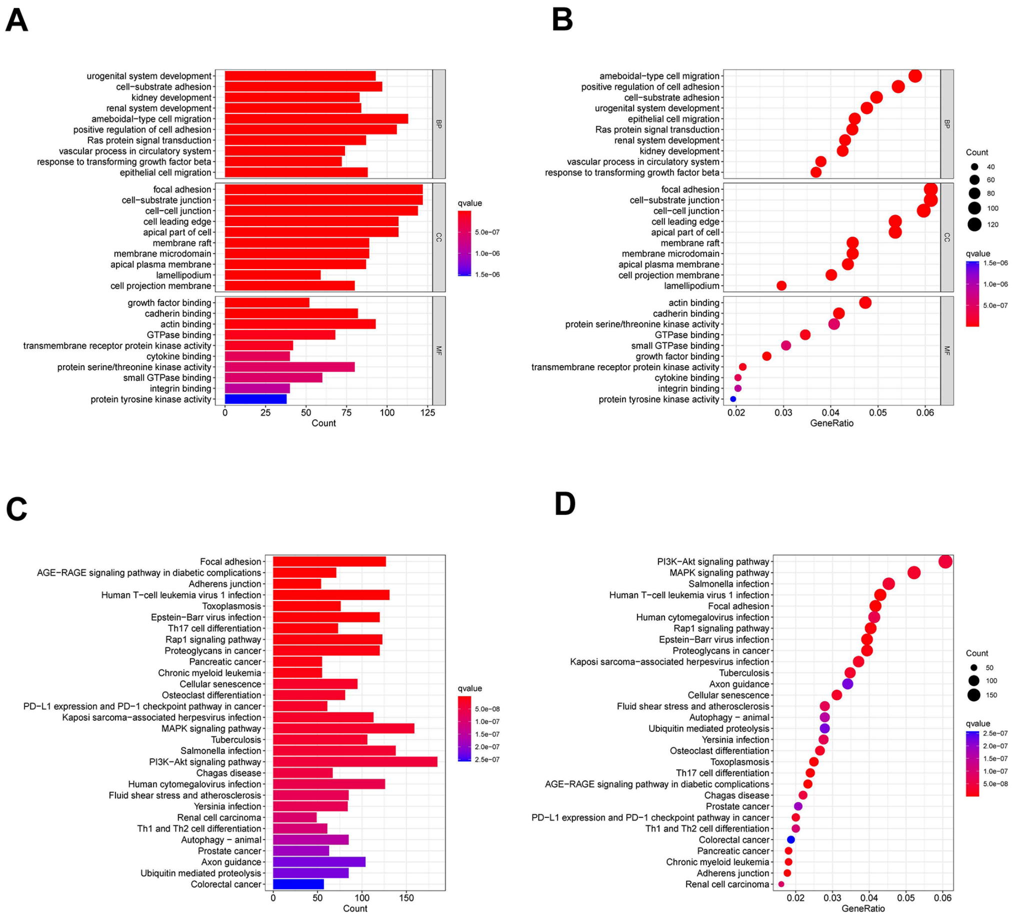

Supplement Figure 2 GO and KEGG enrichment analysis of the differential expressed genes (DEGs) in different clusters. (A), The barplot of GO enrichment analysis for DEGs. (B), The bubble diagram of GO enrichment analysis for DEGs. (C), The barplot of KEGG enrichment analysis for DEGs. (D), The bubble diagram of KEGG enrichment analysis for DEGs.

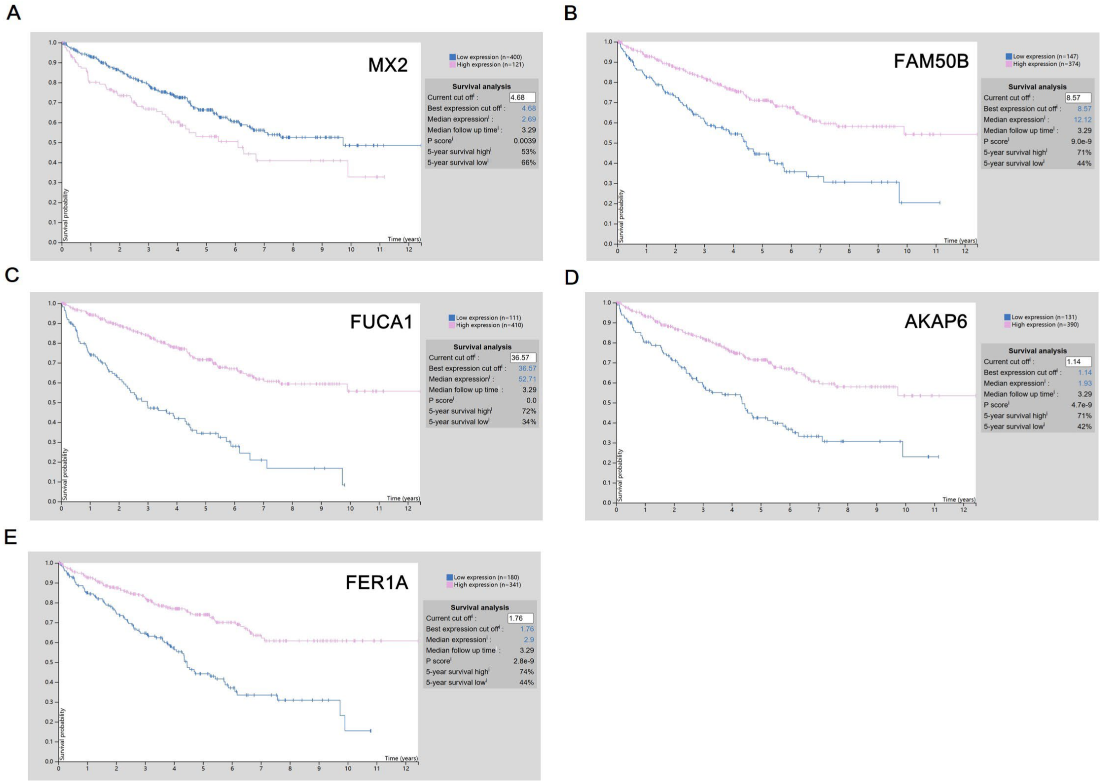

Supplement Figure 3 Kaplan-Meier (KM) curves illustrating the prognosis of patients with varying gene expression levels from the HPA database. (A), MX2. (B), FAM50B. (C), FUCA1. (D), AKAP6. (E), FCER1A.

Supplement Table 1 The differential expressed glycogenes in KIRC

Upregulated genes		Downregulated genes	
A4GALT	FUT7	ABO	GALNT6
ALG1	GAL3ST1	ALG11	GALNT7
ALG13	GAL3ST4	B3GALNT1	GCNT2
ALG14	SLC35D2	B3GALNT2	GCNT4
ALG3	ST3GAL2	B3GALT2	HAS2
ALG5	ST3GAL3	B3GAT1	HS3ST1
ALG6	ST3GAL5	B3GNT8	HS3ST3A1
ALG8	ST6GALNAC4	B4GALNT2	HS3ST3B1
ALG9	ST8SIA1	B4GALNT3	HS3ST5
B3GALT1	ST8SIA3	B4GALT1	HS6ST1
B3GALT6	ST8SIA4	B4GALT6	HS6ST3
B3GAT2	UGCG	C1GALT1C1	MGAT4A
B3GAT3	UGGT2	CHST3	MGAT5
B3GNT4	XTLT2	CHST5	NDST3
B3GNT5	GALNT12	CHST6	NDST4
B4GALNT1	GALNT14	DPM3	POMT1
B4GALNT4	GALNT2	EXTL1	SLC35A2
B4GALT2	GALNT5	EXTL2	SLC35A3
B4GALT3	GBGT1	FUT1	
B4GALT5	GCNT1	FUT10	
B4GALT7	HAS1	FUT2	
CHPF	HAS3	FUT3	
CHPF2	HS2ST1	FUT6	
CHST1	HS3ST2	FUT9	
CHST11	LFNG	GAL3ST2	
CHST12	MFNG	GAL3ST3	
CHST13	MGAT1	ST3GAL4	
CHST14	MGAT2	ST3GAL6	
CHST15	MGAT3	ST6GAL1	
CHST2	MGAT4B	ST6GALNAC2	
CHST7	MGAT5B	ST6GALNAC3	
CHST8	NDST1	ST6GALNAC5	
CHSY1	NDST2	ST3GALNAC6	
CHSY3	OGT	ST8SIA2	
CSGALNACT2	POFUT2	ST8SIA5	
DPM2	POMGNT1	ST8SIA6	
EXT2	RFNG	UGT8	
FKTN	SLC35A1	UST	
FUT11	SLC35B1	GALNT11	
FUT4	SLC35C1	GALNT3	
